# Clinical profiles and treatment outcomes of dental rehabilitation in patients treated under general anesthesia: a comparison between healthy and special healthcare needs children

**DOI:** 10.3389/fped.2025.1550317

**Published:** 2025-04-17

**Authors:** Amal Al-Khotani, Hossam Ajabnoor, Renad Koshak, Shahad A. Alshehri, Dalia E. Meisha

**Affiliations:** ^1^ Dental Department, East Jeddah Hospital, Ministry of Health, Jeddah, Saudi Arabia; ^2^ Division of Anesthesiology and Intensive Care, Department of Surgery, Faculty of Medicine, University of Jeddah, Jeddah, Saudi Arabia; ^3^ Department of Dental Public Health, Faculty of Dentistry, King Abdulaziz University, Jeddah, Saudi Arabia

**Keywords:** general anesthesia, pediatric dentistry, dental rehabilitation, disabled children, hospital dentistry, dental care for children

## Abstract

**Background:**

The dental treatment of pediatric patients under general anesthesia is considered one of the most important behavioral management techniques that parents have accepted. The aim of this study is to compare the clinical profiles and treatment outcomes of healthy patients and special healthcare needs (SHCN) pediatric dental patients who underwent a full dental rehabilitation under general anesthesia (DRGA).

**Methods:**

This study utilizes a retrospective cross-sectional design. Records were reviewed for all pediatric patients referred to the dental clinic from December 2020 to June 2023 and placed on the waiting list for DRGA. The data collected included demographics, medical history, types of dental treatment performed, medications prescribed, and admission/discharge details. Statistical analyses included chi-square, Fisher's exact, Mann–Whitney *U*, Kruskal–Wallis tests, as appropriate, and logistic regression.

**Results:**

This study involved 378 pediatric dental patients treated under general anesthesia (GA), 46.3% were classified as healthy, while 53.7% had SHCN. The SHCN group was significantly older (mean age 6.6 ± 2.7 years vs. 5.1 ± 1.6 years, *p* < 0.0001) and required more extensive treatments, including extractions (*p* < 0.0001), longer hospital stays (mean: 5.9 vs. 0.9 days, *p* < 0.0001), and more frequent preoperative consultations (*p* < 0.0001). The group with children younger than 6 years had a higher proportion of healthy patients (73.9%), received more conservative treatment such as stainless-steel crowns and pulpotomies (*p* < 0.0001), and had shorter hospital stays by 1.3 days compared to the 6–14-year-old group. SHCN status was a strong predictor of admission after DRGA compared to healthy ones (OR: 59).

**Conclusion:**

This study highlights the distinct differences in the clinical profiles and treatment outcomes of healthy patients and SHCN pediatric patients undergoing DRGA, with the latter patients requiring more complex care and experiencing extended hospitalization. These findings underscore the importance of tailoring dental treatment plans to the unique needs of pediatric patients to optimize outcomes.

## Introduction

Medically necessary care (MNC) is the practical, crucial, and optimal service and follow-up care obtained by qualified healthcare providers in order to diagnose and treat any condition, disease, injury, or malformation (congenital or developmental). MNC also involves all supportive services that help improve oral care quality, including sedation, GA, and surgical services. Dental care is a MNC that should directly and positively improve patients' general health and quality of life by minimizing or eliminating orofacial pain and dysfunction to restore esthetics and functions in the oral environment. For pediatric patients, the delivery of MNC in dental settings should include behavioral guidance or management (1).

Behavioral management is the optimal communication and education between the dentist/dental team, the child, and the parents while providing safe dental care. According to the American Academy of Pediatric Dentistry (AAPD), one of the behavioral guidance goals is to provide quality dental treatment safely and comfortably. This, in turn, helps the child have a positive attitude and builds a good relationship with the child during dental treatment. When the dentist fails to control the child's behavior, AAPD suggests several behavioral management techniques, including DRGA (2).

DRGA is a significant pharmacological behavioral guidance modality in pediatric dentistry. It provides dental treatment under optimal conditions, thereby aiming to ensure ideal outcomes (3). DRGA is particularly effective for healthy and medically compromised patients and in cases of urgent or comprehensive treatment, young or uncooperative children with extensive caries, or inadequate parental compliance and cognitive immaturity or disability (2, 4). Practical principles should be applied in customizing anesthetic care for the unique needs of pediatric patients to ensure their safety, comfort, and the best possible outcomes (5). This calls for a detailed assessment of the child's medical history and current health status in order to apply a suitable individualized anesthetic plan (6). It also guarantees relaxation for dental practitioners during dental treatment. This way of evaluation would raise the quality of care and minimize recall visits, allowing dentists to help their pediatric patients get the best outcome.

Other studies have shown that children with health and special needs who underwent DRGA reported better school performance, more social interaction, and increased happiness (3, 7). Faheem and coworkers documented the increased oral-health-related quality of life (OHRQoL) after four weeks for children who underwent DRGA and their families' subsequent lives (8). Additional studies reported high parental satisfaction due to immediate pain relief and improved oral health after the operation (9, 10).

Although numerous international studies have documented the clinical characteristics and outcomes of pediatric patients treated under general anesthesia (7, 8, 11), limited research has been conducted in Saudi Arabia (11, 12). The increasing demand for DRGA services in Saudi Arabia, particularly among children with special healthcare needs (SHCN), highlights the need to better understand how the clinical profiles and treatment outcomes of these patients differ from healthy children. Such insights are crucial to improve care delivery, optimize resource allocation, and ensure equitable access to DRGA for all pediatric patients.

The current study aims to compare the clinical profile and treatment outcomes between healthy and SHCN pediatric dental patients who underwent DRGA.

## Methods

This is a cross-sectional retrospective study of the pediatric patients at East Jeddah Hospital (EJH), Jeddah, Saudi Arabia. The Institutional Review Board (IRB) at the General Directorate of Health Sector, Jeddah, Saudi Arabia, approved this study (approval number: A01632). This study followed the guidelines of the Declaration of Helsinki.

### Participants

**Figure 1:**
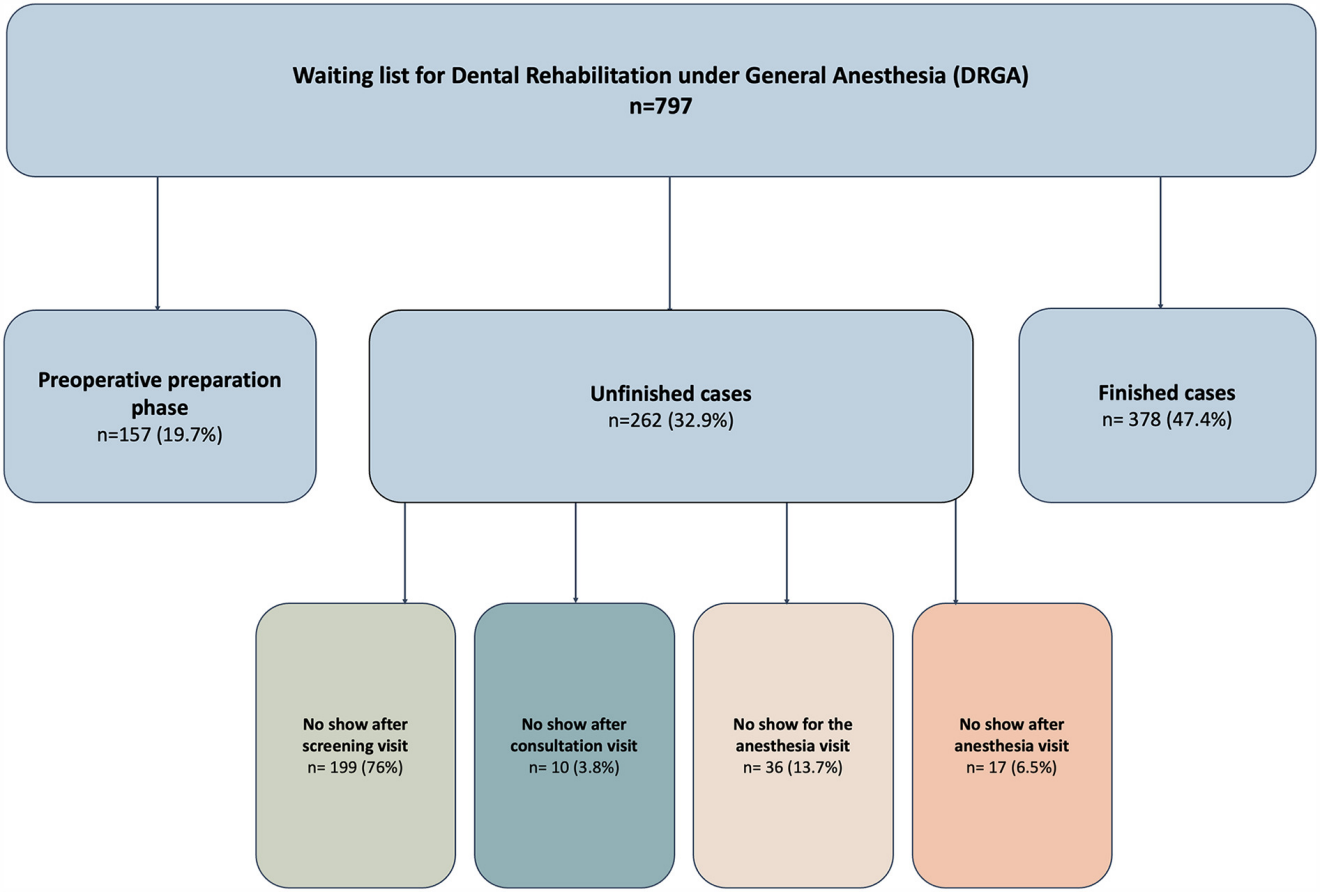
Pediatric patients on the waiting list for Dental Rehabilitation under General Anesthesia (DRGA).

The current study included all pediatric patients referred to the pediatric dental clinic from December 2020 to June 2023 and placed on the waiting list for DRGA. This period included all probable patients from the start of the DRGA at EJH until the data collection time. Figure 1 shows the pediatric patients' flowchart for DRGA.

### Data collection

Cases were obtained from the operation list for pediatric patients who underwent DRGA at the pediatric dentistry division in EJH. The data collection form was designed and piloted prior to the initiation of this study. Training and calibration sessions were conducted for the two data extractors to standardize the extraction process and minimize errors and ensure consistency. The senior principal investigator (PI) systematically reviewed the extracted data to verify its quality. Collected data included demographics (age, gender), medical history, type of dental treatment performed, and admission/discharge details. Patients were excluded if their records were incomplete (e.g., missing key demographic, medical, or treatment data), or if they experienced medical condition that led to the termination of the planned DRGA.

#### 1. Screening process for pediatric patients to the dental operation list at EJH

EJH is a Ministry of Health (MOH) tertiary care hospital located in Jeddah City in the western region of Saudi Arabia. EJH receives referrals for medically compromised pediatric patients from the pediatric department and pediatric dental clinics from other MOH hospitals in Jeddah City. The following is the screening protocol for all patients at the pediatric dental clinic:

1. *Screening visit:* The purpose is to assess the pediatric patient's general and oral health and evaluate the patient's behavior. When the child's cooperation is questionable, an appointment is given at the pediatric dental clinic to assess the child's cooperation.
2. *First dental visit:* The child's behavior is assessed in this treatment visit. When the child is shown to be uncooperative, a discussion is offered to the parents about the DRGA and the dental treatment plan. Then, the child's name is placed on the waiting list if the parents agree.

#### 2. Pediatric patient preparation before the operation

In the preoperative workup for a patient before surgery, the pediatric dental Operation Room (OR) team will perform a thorough assessment, including dental imaging, blood tests, and the operation order in the electronic medical record (EMR) as follows:

(1) *Healthy patients*: All required blood tests should be ordered as a prerequisite from the anesthesia consultant. Then, the patient will schedule a consultation with an anesthesiologist.
(2) *Special healthcare needs patients:* According to the patient's medical condition, a referral appointment with the pediatric consultant is ordered for clearance and to confirm if there are any pre- or post-operative instructions/precautions for the dental operation. Then, the patient is scheduled for blood work and an appointment with the anesthesiologist.

Finally, the anesthesiologist will determine whether the surgery can be performed as a day case or if admission for pre- or postoperative monitoring in the hospital is deemed necessary. The anesthesiologist might recommend the pediatric intensive care unit (PICU) in some critical cases. With the anesthesiologist's evaluation, the surgery schedule date and time are based on the patient's availability, operating room availability, and the pediatric dentist's schedule. After the completion of the DRGA, a follow-up appointment at the pediatric dental clinic is made for one week later as part of the patient's continuation of care.

### Statistical analyses

Statistical analyses were done using IBM SPSS Statistics (Version 24; Armonk, NY: IBM Corp.). The participants were categorized into two groups according to their medical condition: healthy patients and SHCN patients, according to the definition of the AAPD (13). Continuous variables were not normally distributed according to the Shapiro–Wilco test (*P* value <0.001). Bivariate analyses were done using the chi-square test or Fisher's exact test, as appropriate for categorical variables, the Mann–Whitney *U* tests, and the Kruskal–Wallis Tests for continuous variables.

Multivariate logistic regression was used to determine the predictors of being admitted to the hospital versus same-day discharge after DRGA. Variables included in the regression model were age, gender, number of procedures, and health status (healthy vs. SHCN). All tests were performed at a 0.05 level of significance.

## Results

**Figure 2:**
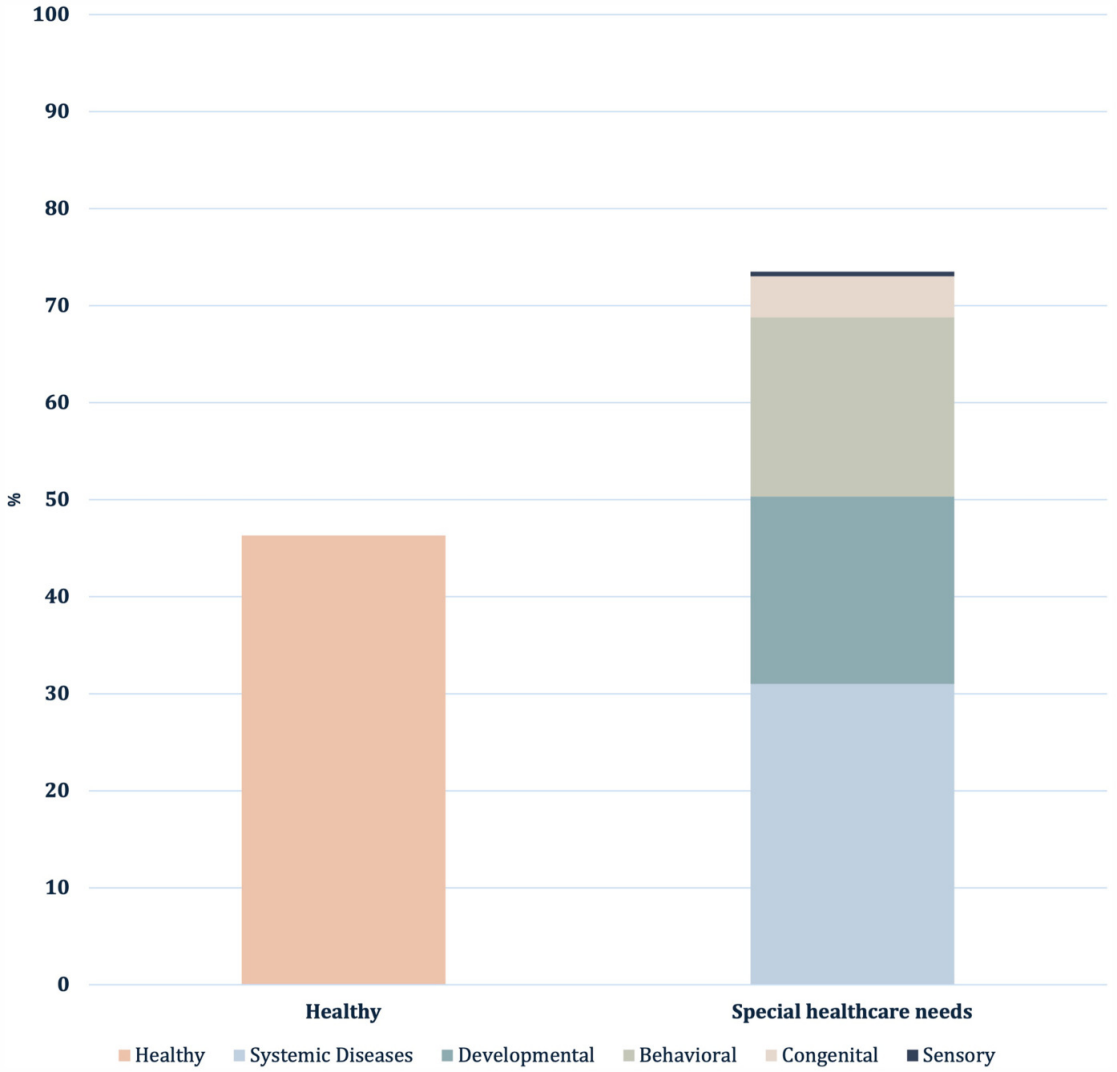
The proportion of healthy and special healthcare needs patients in the study sample.

A total of 378 pediatric dental patients treated under general anesthesia (GA) were included in this study. The demographic characteristics showed that 55.6% of the patients were male and 44.4% were female. The mean age of the patients was 5.9 ± 2.4, with a range of 2–14 years. Less than half of the children were classified as healthy patients (46.3%), and the rest were categorized as SHCN patients (Figure 2). In the behavioral disorders cases, 80% were Autistic Spectrum Disorder (ASD) cases. The most common systemic diseases were neurological (17.2%) and hematological (6.9%). Moreover, 18.8% (*n* = 22) of the children diagnosed with a systemic disease had at least one behavioral problem (ASD or Attention-Deficit Hyperactivity Disorder [ADHD]). Dental procedures were sometimes combined with other procedures: 10.1% of cases with maxillofacial surgery, 2.4% with ENT, and 0.3% with general surgery.

When stratified by age (Table 1), children younger than 6 years had a significantly higher percentage of ASA I classifications compared to older children by 21.8% (*p* < 0.0001). Older children had 3 times the odds of being a SHCN patient compared to younger children (*p* < 0.0001). The prevalence of ASD and ADHD was significantly higher in older children compared to younger ones (*p* < 0.0001). Patients in the older age range had significantly more than twice the odds of needing a consultation and being admitted after the dental procedure compared to the younger age group.

**Table 1:** Characteristics of dental cases treated under general anesthesia stratified by age.

Variables	Age Group				Total *n* = 378
	<6 years (*n* = 192)	6–14 years (*n* = 186) (Ref)	Odds ratio (OR)	*P*-value*	
	*n* (%)	*n* (%)			*n* (%)
Gender			1.2	0.3	
Male (Ref)	102 (48.6)	108 (51.4)			210 (55.6)
Female	90 (53.6)	78 (46.4)			168 (44.4)
ASA-I	142 (73.9)	97 (52.2)	0.4	**<0** **.** **0001**	239 (63.2)
Special healthcare needs:	78 (38.4)	125 (61.6)	3.0	**<0** **.** **0001**	203 (53.7)
Developmental	19 (26)	54 (74)	3.7	**<0** **.** **0001**	73 (19.3)
Congenital	4 (25)	12 (75)	3.2	**0** **.** **04**	16 (4.2)
Behavioral	23 (32.9)	47 (67.1)	2.5	**0** **.** **001**	70 (18.5)
Autistic Spectrum Disorder (ASD)	16 (8.4)	40 (21.5)		**<0** **.** **0001**	56 (14.9)
Attention-Deficit Hyperactivity Disorder (ADHD)	12 (6.3)	23 (12.4)		**0** **.** **04**	35 (9.3)
Systemic	40 (34.2)	77 (65.8)	2.7	**<0** **.** **0001**	117 (31)
Sensory	0	2 (100)	−	0.2	2 (0.5)
Consultation before anesthesia appointment	44 (36.4)	77 (63.6)	2.4	**<0** **.** **0001**	121 (32.1)
Consultation after anesthesia appointment	6 (27.3)	16 (72.7)	2.9	**0** **.** **02**	22 (5.9)
Admission type				**0** **.** **001**	
Ward admission	18 (31)	40 (69)	2.7		58 (15.7)
Day admission (Ref)	171 (54.8)	141 (45.2)			312 (84.3)

Bold values denote statistical significance (α = 0.05).

* Chi-square test or Fisher's exact test, as appropriate.

Table 2 compares healthy patients and those with SHCN who had DRGA. SHCN patients were significantly older than healthy patients; their mean ages were 6.6 ± 2.7 and 5.1 ± 1.6, respectively. SHCN patients waited significantly less from decision to admission and had more extended hospital stays than healthy ones. SHCN patients were more likely to need consultations before (OR: 121.8) and after the anesthesia appointment (OR: 20.2) compared to healthy patients with (*p* < 0.0001). Also, patients with SHCN were more likely to be admitted to the hospital (OR: 68.2) than healthy patients.

**Table 2:** Characteristics of healthy and special healthcare needs patients who had dental rehabilitation under general anesthesia.

Variables	Healthy (*n* = 175)	Special healthcare needs (*n* = 203)	*P*-value*	Total
Age			**<0** **.** **0001**	
Mean ± SD (Min–Max)	5.1 ± 1.6 (2-12)	6.6 ± 2.7 (2-14)		5.9 ± 2.4 (2-14)
Decision to admission (days)				
Mean ± SD	170.1 ± 214.0	152.8 ± 239.2	**0** **.** **03**	160.8 ± 227.7
Median	88	24		50
IQR	282	265		274
Admission to discharge (days)				
Mean ± SD	0.9 ± 11.0	5.9 ± 21.2	**<0** **.** **0001**	3.5 ± 17.4
Median	0	0		0
IQR	0	1		0

	*n* (%)	*n* (%)	*P*-value^†^	*n* (%)
Consultation before anesthesia appointment	2 (1.7)	119 (98.3)	**<0** **.** **0001**	121 (32.1)
Consultation after anesthesia appointment	1 (4.5)	21 (95.5)	**<0** **.** **0001**	22 (5.9)
Admission type			**<0** **.** **0001**	
Ward admission	1 (1.7)	57 (98.3)		58 (15.7)
Day admission	170 (54.5)	142 (45.5)		312 (84.3)

Bold values denote statistical significance (α = 0.05).

* Mann–Whitney *U* tests.

^†^ Chi-square tests or Fisher's exact test, as appropriate.

The mean number of dental procedures performed under general anesthesia was comparable across age groups and health status categories. However, the type of dental treatments provided under GA showed significant variations based on age and health status, as shown in Table 3. Younger patients (<6 years) had a smaller number of primary and permanent teeth extractions and restorations than older children. Conversely, stainless steel crowns (SSC) and pulpotomies were more commonly performed among younger children (*p* < 0.0001). However, SHCN patients had fewer SSCs compared to healthy patients (*p* < 0.0001) and underwent fewer pulpotomies (*p* = 0.001). Permanent tooth extractions were commonly done among SHCN patients (*p* < 0.0001).

**Table 3:** The type of dental treatment received under GA is stratified by age and health status.

Variables	Age			Health status			Total
	<6 years	6–14 years	*P*-value*	Healthy	Special healthcare needs	*p*-value*	
Number of procedures							
Mean ± SD (Min–Max)	12.93 ± 4.23 (0–22)	12.16 ± 4.86 (0–22)	0.2	12.60 ± 4.62 (0–21)	12.51 ± 4.52 (0–22)	0.5	12.55 ± 4.56 (0–22)
Extraction of primaries			**0** **.** **009**			0.8	
Mean ± SD (Min–Max)	4.39 ± 3.53 (0–21)	5.37 ± 3.86 (0–17)		4.72 ± 3.39 (0–14)	5.00 ± 3.99 (0–21)		4.87 ± 3.72 (0–21)
Median	4	5		4	4		4
Restoration			**0** **.** **003**			0.2	
Mean ± SD (Min–Max)	3.56 ± 2.62 (0–13)	4.35 ± 2.85 (0–12)		3.71 ± 2.53 (0–11)	4.16 ± 2.93 (0–13)		3.95 ± 2.76 (0–13)
Median	3	4		4	4		4
Stainless steel crowns (SSC)			**<0** **.** **0001**			**<0** **.** **0001**	
Mean ± SD (Min–Max)	3.61 ± 2.09 (0–8)	1.53 ± 1.90 (0–7)		3.00 ± 2.17 (0–8)	2.23 ± 2.27 (0–8)		2.59 ± 2.25 (0–8)
Median	4	0		3	2		2
Pulpotomy			**<0** **.** **0001**			**0** **.** **001**	
Mean ± SD (Min–Max)	1.35 ± 1.40 (0–6)	0.42 ± 0.87 (0–5)		1.10 ± 1.32 (0–6)	0.72 ± 1.17 (0–6)		0.90 ± 1.25 (0–6)
Median	1	0		1	0		0
Extraction of permanent teeth			**<0** **.** **0001**			**<0** **.** **0001**	
Mean ± SD (Min–Max)	0.02 ± 0.29 (0–4)	0.48 ± 1.20 (0–6)		0.07 ± 0.44 (0–4)	0.40 ± 1.14 (0–6)		0.25 ± 0.90 (0–6)
Median	0	0		0	0		0

Bold values denote statistical significance (α = 0.05).

* Mann–Whitney *U* tests.

**Figure 3:**
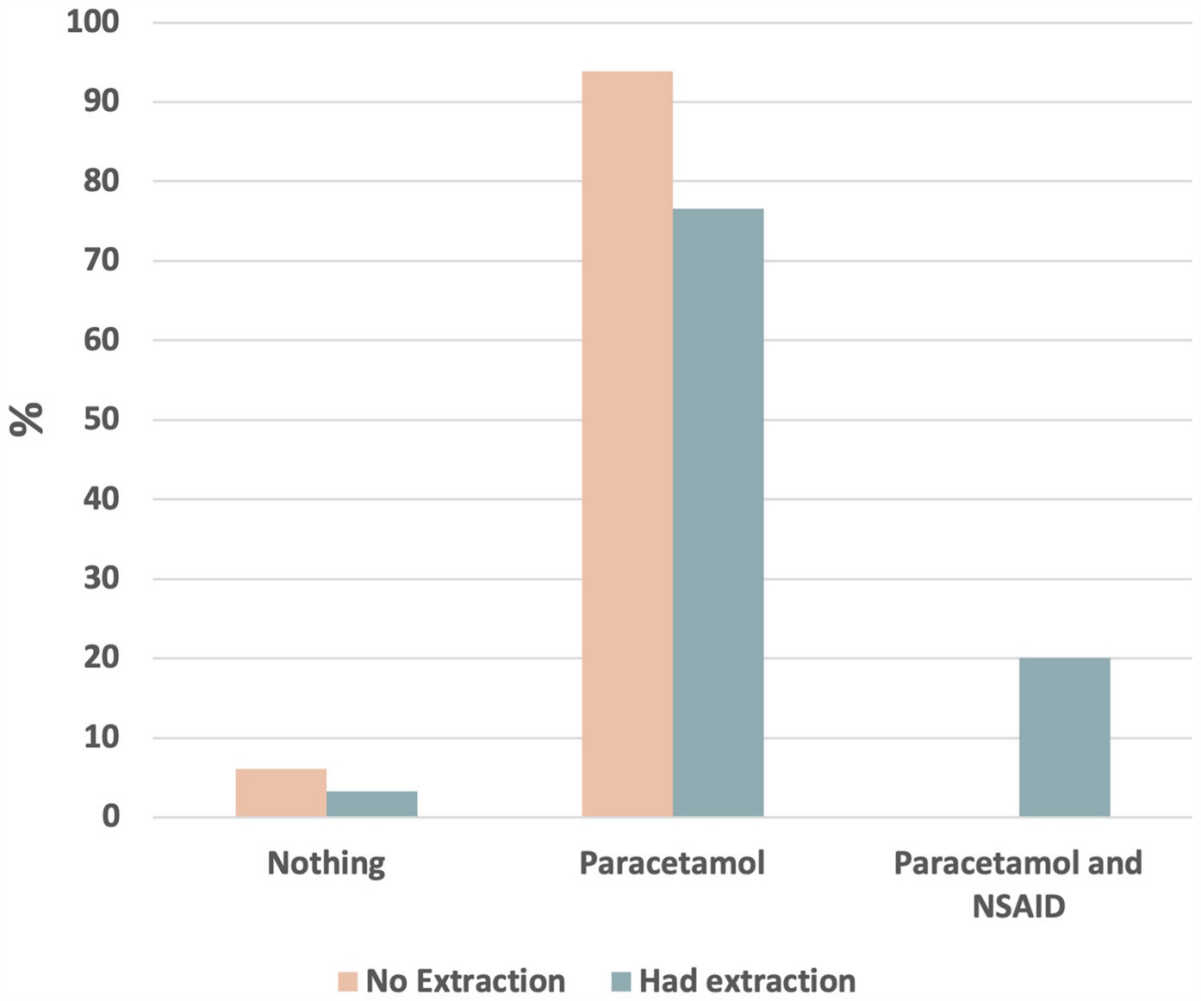
Analgesics prescription in the study sample.

Figure 3 highlights that a patient undergoing extraction had 1.4 odds of being prescribed an analgesic (*P* = 0.02) compared to patients who did not have any extraction. The combination of a nonsteroidal anti-inflammatory drug (NSAID) and paracetamol was only prescribed for patients undergoing extraction.

The multivariate logistic regression analysis identified significant predictors of being admitted compared to same-day discharge after DRGA (Table 4). Children with SHCN had markedly higher odds of being admitted compared to healthy children (OR = 59.0, *p* < 0.0001) even after controlling for age and gender. Age was also a significant predictor, with older children more likely to require admission after DRGA (OR = 1.1, *p* = 0.02). In contrast, gender and number of procedures were not significant predictors in the adjusted model.

**Table 4:** Logistic regression model to predict admission to the hospital compared to same-day discharge after dental rehabilitation under general anesthesia.

Variable	Univariate analysis			Logistic multivariate analysis		
	Crude odds ratio	95% CI	*p*-value	Adjusted odds ratio	95% CI	*p*-value
Health status			**<0** **.** **0001**			**<0** **.** **0001**
- Healthy	Reference			Reference		
- Special healthcare needs	68.2	9.3–499.0		59.0	8.0–436.6	
Age	1.3	1.2–1.4	**<0** **.** **0001**	1.1	1.0–1.3	**0** **.** **02**
Gender			0.9			0.3
- Male	Reference			Reference		
- Female	1.0	0.5–1.7		1.4	0.7–2.6	
Number of dental procedures	1.0	1.0–1.1	0.6	1.0	1.0–1.1	0.3

Bold values denote statistical significance (α = 0.05).

## Discussion

Understanding the clinical profile of pediatric patients undergoing DRGA has significant implications for the future treatment and handling of distinctive pediatric patients with behavioral and developmental disabilities. This study, therefore, aims to contribute to this crucial area of research.

The current study showed that more than half of the patients referred to as being treated under GA had at least one medical problem. This frequency is higher than that of Tsai et al. who reported about a quarter of the children who underwent DRGA were medically ill (14). This difference can be attributed to EJH being the main referral hospital for SHCN children from all MOH hospitals in Jeddah City. Furthermore, the present study showed that the most common medical conditions were neurological, followed by hematology disorders. This finding is similar to Baens-Ferrer et al. who reported that the most common medical condition for children who underwent DRGA is a neurological disorder (15). Another study reported that half of the children who underwent DRGA were diagnosed with developmental delay, and more than a quarter had cerebral palsy and seizure disorders (16). This is not surprising since treating patients with neurological problems in the dental clinic can be challenging due to the possibility of a sudden movement by a patient or a patient not being capable of full cooperation. This fact can explain the high percentage of children with neurological problems who underwent DRGA in our study like in other studies (10, 14–16).

The present study also showed that about 19% of the children with a diagnosed systemic disease exhibited at least one behavioral problem: ASD or ADHD. This finding is reasonable since one of the indications of DRGA is the lack of cooperation in the dental clinic due to special conditions that jeopardize the child's safety (17). Similarly, another study showed that about one-third of patients with behavioral problems underwent DRGA.

When the medical condition was stratified by age, the present study recorded that more than half of the healthy pediatric patients who underwent DRGA were younger than six. This result aligns with retrospective study results that reported that more than three-quarters of healthy young pediatric patients who underwent DRGA were under six (14). Another study found that young pediatric patients are the most common group receiving dental treatment under GA (18). One reason for this consistency between studies is that uncooperative young patients are considered the second most common indication for dental treatment under GA due to the immaturity of those young children (2). Studies have shown that most children who could cope with dental treatment in the clinic were above the age of six, which is comparable to our findings (14). With this in mind, the current findings confirm the uniform process between the pediatric dentists in our department regarding the behavior management techniques used that follow the AAPD guidelines (2), providing reassuring reliability in the field.

The current study also found that over half of the pediatric patients with medical, behavioral, and developmental problems who underwent DRGA were older than 6. This finding seems rational since the lack of cooperation among children was due to their medical condition, which aligns with many former studies (14, 19). Those studies showed that children with SHCN had higher caries severity compared to healthy ones, especially in their permanent molars (14). This could be due to the reality that parents of medically compromised children become busy with frequent visits to the physician at an early stage (20). In turn, they become aware of their children's oral condition only when their children's general health becomes affected by the oral diseases. It is worth mentioning that dental pain/abscesses will aggravate the children's oral condition and, in turn, worsen their medical condition (21, 22).

The type of dental treatment under GA should be carefully selected, especially for those with uncertain prognoses, in order to ensure the success of this comprehensive procedure (23). Extant studies have reported various treatment modalities used in DRGA, ranging from restorations, SSC, and pulp therapy to tooth extraction (18, 24–27). The same studies recommended encouraging “radical treatment,” meaning that non-restorable and questionable teeth should be extracted even with permanent teeth. The current study demonstrated a statistically significant difference in the type of treatments performed between the younger and older age groups. The mean number of primary and permanent teeth extractions was substantially higher in older children compared to younger ones, consistent with previous studies reporting more extractions and fewer SSCs, and pulpotomies in children over six years old (18, 26, 28). This trend reflects the emphasis of DRGA on ensuring long-term treatment success, often favoring more definitive treatment approaches, particularly for teeth with questionable prognoses (27). Additionally, the higher prevalence of medical comorbidities among older children may further contribute to the increased likelihood of extractions due to poorer overall health with questionable prognosis of teeth restorability (26, 28).

Many studies reported that parents usually ignore the importance of primary teeth and think that permanent molars are primary molars and will be replaced in the future (29, 30). Due to this mistaken belief, young permanent molar tooth structures become seriously damaged and might reach the pulp at an early age. In contrast, Bader et al. recommended that the treatment provided for DRGA is pulpectomy or root canal treatment rather than extraction to save the affected teeth as space maintainers (31). This suggestion does not seem applicable in the current study because the primary aim of DRGA is not only to emphasize the efficiency of dental treatment but also to ensure the long-term prognosis and prevent the need for a second DRGA (32). However, Chen and coauthors stated that pulpectomy is becoming the last choice in dental treatment under GA (18).

Our study found that dental treatment using pulpotomy and SSC restoration was used more with younger patients. This finding is supported by previous studies that reported using SSCs with a higher success rate in the young age group than adhesive restoration (18, 33). The current study showed statistical differences between the old-age and young-age groups in adhesive restorations. However, the mean for adhesive restorations done for the old-age group is higher than that of the young-age group. Adhesive restorations are more prone to failure than SSCs at a young age, where there is a long time before primary dentition exfoliation (33). Similarly, Guidry et al. found that most patients received dental treatments with adhesive restorations; they needed a second DRGA, especially for those with poor oral hygiene (23). Another possible reason could be that the primary teeth in the old age group (>6) will be exfoliated in a few months/years. This supports the notion of using adhesive restorations instead of SSC for patients in late mixed dentition is comparable to the present study (18).

When the type of dental treatment stratified by the medical conditions, the current study showed a significant difference between the healthy and SHCN groups in SSC, pulpotomy, and extractions of permanent teeth treatments. SSC and pulpotomy treatment means were significantly higher in the healthy group than in the SHCN group. At the same time, the mean of extracting both primary and permanent teeth was higher in the SHCN group than in the healthy group. This significance can be attributed to the severity of the medical problem that will, in turn, lead to severe tooth destruction and tooth loss at an early age. Similarly, Forsyth et al. recommended using a more aggressive approach with medically ill children (34). Since a failed restoration can be life-threatening and should involve additional surgical interventions (18, 24), our study used intensive treatment for children with SHCN.

Our study showed significant differences in the waiting time for DRGA between healthy children and those with SHCN. The median time for SHCN was 24 days, compared to 88 days for healthy patients, reflecting the prioritization of medically complex cases. This prioritization is warranted, as delays in dental care for children with SHCN can negatively impact both their oral and overall health, potentially exacerbating existing medical conditions (14, 20). Similarly, Lewis et al. reported that among American pediatric patients, the average waiting time for DRGA was 28 days for those experiencing pain and 71 days for those without pain (35).

In reference to the high prevalence of postoperative pain in pediatric patients after DRGA for about 3 days (36, 37), our study showed an association between the number of extracted teeth and the prescription of combined analgesics (paracetamol and ibuprofen) alternatively for at least 2 days. Similarly, Marshall et al. have proven that this combined management would substantially reduce the pain post-DRGA (38).

This study has several strengths. First, the existing study included all eligible pediatric patients admitted to DRGA at EJH during the study period. Second, this model may provide a representative sample for medically compromised pediatric patients since EJH received referrals for those patients from all of Jeddah City during the study period. Third, the data were extracted from computer systems and paper-based records, which might increase their reliability. However, the retrospective nature of this study might be a limitation. Also, the data can be limited since it represents a single center and may limit the generalizability of our results. Nevertheless, the absence of a comparison group that has not undergone the DRGA could be addressed in future studies to assess the relative effectiveness and risks of this approach. In addition, long-term evaluation of the treatment outcomes would add valuable insight in future research.

## Conclusion

This study highlights differences in the clinical profiles and treatment outcomes of healthy and SHCN pediatric patients undergoing dental rehabilitation under GA. Younger healthy patients typically required more conservative treatments and shorter hospital stays, while SHCN patients were older, required more complex care, and experienced extended hospitalization. Given the growing number of pediatric patients treated under GA, prioritizing timely access to DRGA for SHCN patients is crucial to minimizing complications and improve outcomes. Adopting radical treatment approaches may optimize long-term oral health while enhancing the overall experience for children and their families.

## Data Availability

The raw data supporting the conclusions of this article will be made available by the authors, without undue reservation.
